# Endophyte Microbiome Diversity in Micropropagated *Atriplex canescens* and *Atriplex torreyi* var *griffithsii*

**DOI:** 10.1371/journal.pone.0017693

**Published:** 2011-03-17

**Authors:** Mary E. Lucero, Adrian Unc, Peter Cooke, Scot Dowd, Shulei Sun

**Affiliations:** 1 Jornada Experimental Range, Agricultural Research Service, United States Department of Agriculture, Las Cruces, New Mexico, United States of America; 2 Plant and Environmental Sciences, New Mexico State University, Las Cruces, New Mexico, United States of America; 3 Electron Microscopy Laboratory, New Mexico State University, Las Cruces, New Mexico, United States of America; 4 Research and Testing Laboratories, LLC., Lubbock, Texas, United States of America; 5 Center for Research in Biological Systems, University of California San Diego, La Jolla, California, United States of America; University of Missouri-Kansas City, United States of America

## Abstract

Microbial diversity associated with micropropagated *Atriplex* species was assessed using microscopy, isolate culturing, and sequencing. Light, electron, and confocal microscopy revealed microbial cells in aseptically regenerated leaves and roots. Clone libraries and tag-encoded FLX amplicon pyrosequencing (TEFAP) analysis amplified sequences from callus homologous to diverse fungal and bacterial taxa. Culturing isolated some seed borne endophyte taxa which could be readily propagated apart from the host. Microbial cells were observed within biofilm-like residues associated with plant cell surfaces and intercellular spaces. Various universal primers amplified both plant and microbial sequences, with different primers revealing different patterns of fungal diversity. Bacterial and fungal TEFAP followed by alignment with sequences from curated databases revealed 7 bacterial and 17 ascomycete taxa in *A. canescens*, and 5 bacterial taxa in *A. torreyi*. Additional diversity was observed among isolates and clone libraries. Micropropagated *Atriplex* retains a complex, intimately associated microbiome which includes diverse strains well poised to interact in manners that influence host physiology. Microbiome analysis was facilitated by high throughput sequencing methods, but primer biases continue to limit recovery of diverse sequences from even moderately complex communities.

## Introduction

*Atriplex* is a globally distributed halophyte genus valued for forage, restoration, and remediation potential [1], [2], [3]. Species within the genus are known for complex genetics [4], rapid evolutionary rates [5], and high tolerance to xeric, saline, and contaminated soils [6], [7]. *Atriplex canescens*, a species widely distributed across arid regions of North America, has been noted for high phenotypic diversity commonly attributed to complex genetic patterns resulting from sexual lability and polyploidy [4], [8].

The degree to which microbial associations with *Atriplex* also contribute to phenotypic variation and host adaptation merits deeper investigation [9]. The genus was initially categorized as non-mycorrizal, suggesting an absence of associations with vesicular arbuscular mycorrhizal (VAM) fungi. Later work revealed conditional VAM associations related to the surrounding habitat [10], and even low levels of mycorrhizal colonization were determined to benefit *Atriplex* growth and nutrient uptake [11]. More recently, Barrow et al. recognized and documented systemic *Atriplex canescens* colonization by dark septate endophytic fungi (DSE) [12], [13]. These DSE are not as readily detected in plants as VAM fungi, but are equally capable of influencing plant performance [14]. Many of the various fungi associated with *Atriplex canescens*, are seed borne, and facilitate seedling establishment [15]. These seed borne fungi remain systemically associated with clonal progeny cultivated *in vitro* [16], suggesting a mechanism through which plants and symbiotic fungi may share co-evolutionary pathways in which fungi are vertically transferred from parent to either clonal or sexually produced progeny.

The ability of seed borne systemic endophytes to influence adaptation of both host and progeny to stressed or changing habitats has been examined extensively in cool season grasses [17], [18], [19], where the invasive nature of endophyte-infected fescue, combined with the toxicity of endophyte-produced alkaloids, has resulted in significant economic loss [20]. Despite the negative aspects of endophyte association, interest in the drought hardiness and insect resistance conferred by the endophytes has sparked interest in biotechnological development of endophyte-colonized grass cultivars [21]. Meanwhile, toxic locoweeds (*Oxytropis* sp.), another group of plants known for toxicity to grazing livestock, have also been shown to derive toxicity from compounds produced by endophytes [22]. In locoweeds, the toxin production by the endophyte appears to be enhanced in the presence of a nitrogen fixing bacterium, indicating a complex plant-fungal-bacterial interaction.

While clavicipitaceous fungi are known for producing toxic alkaloids that increase host defense capability [23], non-clavicipitaceous fungi represent a larger array of taxa, have been less thoroughly investigated, and may offer a more diverse range of potential benefits to their hosts [24], [25]. Experiments in which endophyte laden callus tissue of *A. canescens* was used as inoculum for native grasses suggest *Atriplex* associated microbes may confer benefits to alternate host species [26], [27]. Difficulties with detection and monitoring uncultured endophytes, combined with the complexity of the microbial consortium retained in *Atriplex in vitro* have made it difficult to determine which endophytes, if any, are being transferred from the callus inoculum to seedlings [26], [27], prompting a need for a comprehensive analysis of the *in vitro* microbial community.

Compared with traditional capillary sequencing methods, high-throughput pyrosequencing yields more data on the diversity of microbes in different habitats. Using high throughput methods, sequences of nuclear ribosomal small subunit (16S) are widely used to identify bacterial diversity while the small subunit (18S) and the internal transcribed spacer (ITS) sequences are commonly used to characterize fungal communities.

Our objectives were to explore the diversity of systemic endophytes detected in micropropagated lines of two *Atriplex* species using varied techniques in order to maximize detection of cryptic species with potential to benefit the host. The species chosen included *Atriplex canescens* (Pursh) Nutt. (ATCA2), which is broadly distributed across arid western regions of North America from Alberta, Canada to central Mexico, and *Atriplex torreyi* (S. Watson) S. Watson var. *griffithsii* (Standley) G.D. Brown (ATGR2), an isolated subspecies found only within a few discontinuous saline areas of southern New Mexico and Arizona in the United States [28]. Throughout the text, we will use the term *endophyte* to collectively describe phyllosphere microbes that persist asymptomatically in surface disinfested, aseptically maintained plants, without visible growth beyond the plant on culture media.

## Methods

### Plant Materials

*A. canescens* seeds were collected from a single parent plant associated with a stable, *Atriplex* dominated shrub population that has been documented on the USDA-ARS Jornada Experimental Range near Las Cruces, NM (32.67150, -106.71812) since 1858 [29].

*A. torreyi* callus was initiated from a plant located within a population growing along the edge of a dry lake bed west of Lordsburg, NM (32.28239,-108.86870). Viable seeds were unavailable at the time of collection. Voucher specimens of both species were deposited in the Range Science Herbarium at New Mexico State University (Las Cruces, NM.). *A. torreyi* clones, regenerated as described by Reyes-Vera et. al., were donated to the Rio Grande Botanic Garden in Albuquerque, NM [30].

### Isolation of Culturable Microbes

*A. canescens* seeds (n = 90) were excised from the utricles as described above and surface disinfested overnight in 15% hydrogen peroxide. Disinfested seeds (n = 10) were plated on media with varied nutritional and salinity content as follows: Nutrient Agar (Difco™, Becton, Dickinson, and Company, USA) Potato Dextrose Agar (PDA, Difco™, Becton, Dickinson, and Company, USA), 0.1X PDA (3.9 g Difco™ PDA and 13.5 g plant tissue culture grade agar per liter of water), Malt Extract Agar (MEA, Difco™, Becton, Dickinson, and Company, USA), MEA+1.5S (MEA +1.5% Instant Ocean® marine salt), MEA+3.0S (MEA +3.0% Instant Ocean® marine salt), MEA+1.5 NaCl (MEA +1.5% sodium chloride), and MEA +3.0 NaCl (MEA +3.0% sodium chloride), Plates were sealed with a paraffin-based laboratory film and incubated at 26°C in an unlighted area for up to 30 days. Plates were inspected weekly. When detected, approximately 1 mm^2^ samples of fungal hyphae were isolated by aseptically transferring to fresh plates of the same medium, and fresh plates of PDA. Colonies without visible hyphae (bacteria or yeast-like colonies) were streaked onto Nutrient Agar with a sterile inoculating loop, and single colonies were isolated. Isolates growing on PDA and on Nutrient Agar were grouped by morphology into 4 distinct groups. A single representative from each group was selected for rDNA analysis.

### Micropropagation

To minimize interference from external microbes, plant materials were cultivated aseptically, *in vitro*. Shoot induction was carried out as described by Reyes-Vera et al. [30]. Briefly, apical shoots (*A. torreyi*) and whole seeds (*A. canescens*) that had been mechanically excised from the surrounding utricles were surface disinfested by soaking in a 1∶100 solution of Zerotol™, (Biosafe Systems, LLC, Connecticut, USA) in sterile water for 30 min. Disinfested tissues were plated directly onto shoot induction medium (2.41 g^.^L^−1^ Woody Plant Media with vitamins (product no. L449, PhytoTechnology Laboratories®, Kansas, USA), 30 g^.^L^−1^ sucrose, 5 mg^.^L^−1^ 6-(γ-γ-dimethylallylamino) purine and 0.8% plant tissue culture grade agar (pH 5.6+/−0.05)). Plates with visible microbial growth were eliminated from the populations. After 30 days, clearly visible shoot primordia with no visible signs of microbial presence were transferred to culture boxes with vented lids and fresh medium. Callus associated with shoot clusters was transferred to callus medium (2.41 g^.^L^−1^ Woody Plant Media with vitamins (product no. L449, PhytoTechnology Laboratories®, Kansas, USA), 30 g^.^L^−1^ sucrose, 0.75 mg^.^L^−1^ picloram 2 mg^.^L^−1^ 6-benzylaminopurine and 0.8% plant tissue culture grade agar (pH 5.6+/−0.05)). Shoots and calli were incubated at 28±1°C under continuous fluorescent light (14–18 µmol m-2⋅s-1) and were aseptically subcultured to fresh medium every 30 days.

### Seed preparation for microscopic analysis of uncultivated, seed borne microbes

Whole *A. canescens* seeds (with bracts and utricles intact) and seeds that had been excised from the utricles were heavily surface disinfested by vortexing for 1 min in a solution of 100% ethanol +0.01% polysorbate 20. Seeds were rinsed 2X in sterile water, then submerged in a 1∶50 Zerotol™ (Biosafe Systems, LLC, Connecticut, USA), vortexed for 10 min, and stored overnight in a sealed centrifuge tube at 4°C. Next, disinfested seeds were transferred to Petri dishes containing Murashigie and Skoogs medium [31] and incubated for 1 week at 37°C.

### Light microscopy

Seeds excised from the utricles were cleansed thoroughly by vortexing for 10 minutes in a 1∶50 solution of (Zerotol™, Biosafe Systems, LLC, Connecticut, USA) in water, followed by two rinses with water. Seeds were either prepared for microscopy immediately by embedding in Tissue Tek®, O.C.T compound (Sakura Finetek USA, Inc., Torrance, California, USA) and freezing at −22°C for 6–16 h, or were allowed to germinate for 48 hours on water agar at 28±1°C under continuous fluorescent light (14–18 µmol m-2⋅s-1) prior to embedding. Leaves and stems of regenerated shoots were soaked overnight in 15% glycerol, then embedded in O.C.T compound, and frozen as above. Tissues were sliced into 2–8 µm sections using a Microm HM520 cryostat (Thermo Fisher Scientific, Inc., Massachusetts, USA), and transferred to Polysine® coated microscope slides (Thermo Fisher Scientific, Inc., Massachusetts, USA). O.C.T. compound was removed and tissues were electrostatically bound to the slides by rinsing 2 times with sterile, deionized water at 65°C for 1 min. Excess water was removed with a transfer pipette and sections were circled with a hydrophobic pen (PAP Pen, Electron Microscopy Sciences, Pennsylvania, USA), and allowed to air dry for no more than 1 h. Sections within the circled area were stained by covering with a drop of lactophenol cotton blue (BD Diagnostic Systems, USA, Product number 261188) for wet mounts, or a drop of either trypan blue stain (0.05% trypan blue, 50% glycerol, and 0.05% hydrochloric acid in water.), or 0.5% toluidine blue in water for permanent mounts. Permanent mounts were heated to 65°C for 1 min. Stain solution was removed with a transfer pipette, and slides were rinsed with sterile, deionized water until no dissolved stain was visible in the rinse solution. Tissues were air dried as above and mounted with cover slips. Tissues were examined with bright field microscopy on a Zeiss Axiovert 200 M microscope.

### Confocal Microscopy

Leaves of micropropagated plants were transected at mid-length with a stainless steel razor blade and the cut surfaces were mounted on a glass bottom culture dish (Mat-Tek Corp., Ashland, MA) and pre-incubated according to the manufacturer's instructions with the ‘live’ viability dye in the kit (L-7012, Invitrogen, Corp., Carlsbad, CA) followed by immersion in a fixative solution, 2.5% glutaraldehyde in 0.1 M imidazole-HCl, pH 7.2) before imaging with a spectral confocal microscope (Leica Microsystems, Exton, PA) equipped with a long working distance 20X objective lens. Green fluorescence (500–540 nm) was collected in one channel, autofluorescence (580–680 nm) was collected in a second channel and maximum projection images of 20–30 µm tissue slabs were overlaid in order to search visually for microbes within the leaf microstructure.

### Electron Microscopy

For scanning electron microscopy, fresh tissues (within 1 h of harvesting) were placed in the vacuum chamber of a Hitachi S3200N scanning electron microscope under variable pressure mode and examined at varied levels of magnification.

### DNA isolation, amplification, cloning, and sequencing

Samples of Atriplex callus and of fungal isolates representative of each morphotype, were ground under liquid nitrogen in a mortar and pestle and either temporarily stored at −80°C or immediately transferred into bead tubes for DNA extraction using PowerPlant® DNA isolation kits (MoBio Laboratories, Inc., Carlsbad, California, USA) according to the manufacturer's protocol. To reduce potential for contamination of PCR reactions, amplifications were carried out in a PCR hood using dedicated, UV resistant pipettors with factory sterilized barrier tips. The hood and pipettors were routinely cleaned with DNA Erase™ (MP Biomedicals, LLC, Solon, Ohio) followed by UV treatment prior to use. Fungal rDNA internal transcribed spacer (ITS) regions of genomic DNA were amplified using the primer pairs and annealing temperatures listed in Table 1. Initial PCR products were either directly sequenced or cloned into a pCR2.1 cloning vector using the TA Cloning® kit (Invitrogen, Carlsbad, California, USA) according to the manufacturer's protocol. Capillary sequencing was performed by a commercial laboratory (Functional Biosciences, Inc., Madison, Wisconsin, USA).

**Table 1:** Primers and annealing temperatures (TA) utilized for amplification of endophyte DNA from total DNA extracted from micropropagated plant tissues and/or isolates.

Target	Primer	Sequence	TA	GenBank or CAMERA Accessions
16S	Gray28F [75]	GAGTTTGATCNTGGCTCAG	52	CAM_PROJ_AtriplexMicrobiome_SMPL_ATGR_16S
	Gray519R [75]	GTNTTACNGCGGCKGCTG		CAM_PROJ_AtriplexMicrobiome_SMPL_ATCA-J_16S
	ITS1F [50]	CTTGGTCATTTAGAGGAAGTAA	55	*HM596872, HM596871, HM596868, HM596870,* **FJ601837**, HM596874, HM596873
	ITS4 [52]	TCCTCCGCTTATTGATATGC		
	NSI1 [42]	GATTGAATGGCTTAGTGAGG	65	HM596876
	NLB4 [42]	GGATTCTCACCCTCTATGAC		
	ENDOITSF	AAGGTCTCCGTAGGTGAAC	48.5	CAM_PROJ_ATRIPLEXMICROBIOME_SMPL_T524-EndoITSFR, HM596875*, HM998754*
	ENDOITSR	GTATCCCTACCTGATCCGAG		
	58A1F [42]	GCATCGATGAAGAACGC	58	CAM_PROJ_ATRIPLEXMICROBIOME_SMPL_T524-NLB4
	NLB4 [42]	GGATTCTCACCCTCTATGAC		
	ITS5 [52]	GGAAGTAAAAGTCGTAACAAGG	53	FJ601833*
	ITS4 [52]	TCCTCCGCTTATTGATATGC		
	ITS1F [50]	CTTGGTCATTTAGAGGAAGTAA	62	no product
	ITS4B [50]	CAGGAGACTTGTACACGGTCCAG		
	ITS5 [52]	GGAAGTAAAAGTCGTAACAAGG	60.1	FJ601837, FJ601839*
	ITS4A [76]	CGCCGTTACTGGGGCAATCCCTG		
SSU	NS3 [52]	GCAAGTCTGGTGCCAGCAGCC	62	**FJ601841**
	NS4 [52]	CTTCCGTCAATTCCTTTAAG		
	NS3 [52]	GCAAGTCTGGTGCCAGCAGCC	59	FJ601842*, *HM195297, HM195296, HM195295, HM596869*
	NS8 [52]	TCCGCAGGTTCACCTACGGA		
	funSSUF	TGGAGGGCAAGTCTGGTG	52	CAM_PROJ_AtriplexMicrobiome_SMPL_ATGR_SSU,
	funSSUR	TCGGCATAGTTTATGGTTAAG		CAM_PROJ_AtriplexMicrobiome_SMPL_ATGR_SSU, CAM_PROJ_AtriplexMicrobiome_SMPL_ATCA-J_SSU

### Bacterial (bTEFAP) and fungal (fTEFAP) tag-encoded FLX amplicon pyrosequencing

To identify diverse microbes missed by cloning, genomic DNA aliquots were subjected to semi-quantitative detection and identification methods for bacteria and fungi based on the Roche 454 Titanium pyrosequencing platforms (Roche, Nutley, New Jersey) The materials and custom approaches for both methods have been previously described but are summarized with references below.

### bTEFAP

Each sample was analyzed using bacterial tag-encoded FLX amplicon pyrosequencing (bTEFAP) to determine the bacterial populations present [32], [33], [34], [35], [36]. bTEFAP and data processing were performed as described previously [33], [35], except that the bTEFAP was based upon the Titanium sequencing platform rather than FLX (Roche Applied Science, Indianapolis, IN). Titanium differs in that it generates average read lengths of 400 bp rather than 250 bp generated by the previous FLX chemistry. The primers utilized also differed from those previously reported. The proprietary primers utilized herein extended from 27F numbered in relation to *Escherichia coli* 16s ribosome gene (Research and Testing Laboratory, Lubbock, TX). Finally, rather than the double PCR utilized in the previous methods, only a single step reaction (35 cycles) was utilized and 1U of HotStar HiFidelity Polymerase was added to each reaction (Qiagen). Raw data from bTEFAP was screened and trimmed based upon quality scores and binned into individual sample collections. Sequence collections were then depleted of short reads (<300 bp) using B2C2 http://www.researchandtesting.com/B2C2.html). Bacterial taxa were identified using BLASTn comparison to a curated, high quality bacterial 16S database derived from NCBI. Relative percentages of bacterial sequences with the highest sequence similarity to a single genus or higher level taxonomic ID were determined for each individual sample (Table 2). Sequences that aligned best to cyanobacteria, but had percent identity scores less than 90% were tentatively identified as organelles.

**Table 2:** Bacteria detected in *A. canescens* (ATCA2) *A. torreyi var griffithsii* (ATGR2) using bTEFAP analysis of DNA extraced from micropropagated callus.

Phylum	ID	ATCA2	ATGR2
Bacteroidetes	Bacteroides	0	0.01
Viridiplantae	organelles	68.3	99.96
Firmicutes	Bacillaceae	0.05	0
Firmicutes	Geobacillus	0.01	0
Firmicutes	Staphylococcus	19.14	0.01
Firmicutes	Clostridium	0	0.01
Firmicutes	Sporobacter	0	0.01
Proteobacteria	Caulobacter	0.02	0
Proteobacteria	Beijerinckia	12.45	0
Proteobacteria	Rhizobiales	0.01	0
Proteobacteria	Sphingomonas	0.01	0
Proteobacteria	Escherichia	0	0.01

The ID column lists the genus or the lowest taxonomic classification in which the sequences could be placed. Sequences with more than 95% similarity to sequences representing more than one genera are identified at the most precise taxonomic level that encompasses all matching genera. The numeric values under the columns labeled ATCA2 and ATGR2 represent the percent of sequences identified within the indicated callus line which matched each taxonomic ID. Taxa highlighted in bold type were detected in both callus lines.

To verify identification as organelle sequences, quality screened sequences exceeding 300 bp were clustered to consensus sequences representing clusters that were 95% identical over 80% of their length. Clustering was carried out using the CD-HIT program contained within the RAMCAP pipeline at CAMERA [37], [38], [39]. The output contained 32 consensus sequences, which were classified using the RDP Classifier [40] at the ribosomal database project [41].

### fTEFAP

For fTEFAP analysis, total genomic DNA from *A. canescens* callus was either directly amplified with a proprietary pair of primers targeting the fungal small subunit (SSU) ribosomal region and supplied by Research and Testing Laboratory, LLC (Lubbock, Texas, USA), or amplified using the primer pairs ITSF and ITS4B, ITS1F and ITS4, ENDOITSF and ENDOITSR, or NSI1 and NLB4 (all of which targeted the ITS region) under the conditions illustrated in Table 1. Products amplified with NSI1 and NLB4 were reamplified with 58A1F and NLB4 as described previously to improve selectivity for fungal DNA contaminated with plant sequences [42]. Amplified products were subjected to fTEFAP as described for bTEFAP above, using the same primer pairs used in the initial or nested (58A1F and NLB4) amplification. Results were compared against a curated fungal sequence database maintained by Research and Testing Laboratories, Inc.

### Phylogenetic analysis

**Figure 1:**
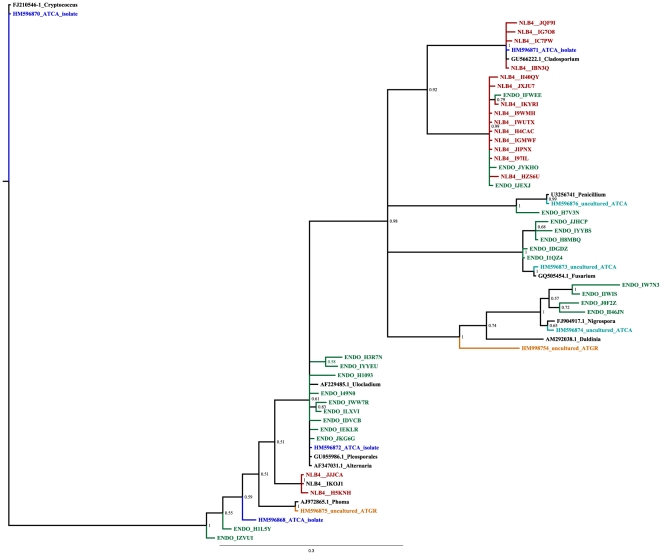
Bayesian analysis of ITS gene sequences from *Atriplex* associated fungi. Sequences were derived from isolates (blue), clones of uncultured fungal sequences extracted from micropropagated *A. canescens* (turquoise) or *A. torreyi* var. *griffithsii* (orange), or consensus sequences of uncultured *A. canescens* fungi obtained from tag-encoded pyrosequencing analysis of PCR products amplified with the primers ENDOITSF and ENDOITSR (green), and nested PCR products amplified as described by Martin and Rygiewicz [42] (red).

Fungal ITS sequences were subjected to phylogenetic analysis to explore similarity between taxa amplified with varied primers and sequenced by conventional and next generation techniques. Clone sequences were initially subjected to pairwise (BLASTn) searches against NCBI and AFTOL (Assembling the Fungal Tree of Life) [43] databases. Sequences with the highest scoring BLASTn matches were added to the analysis. Sequences resulting from fTEFAP analysis (Table 1, accessions CAM_PROJ_ATRIPLEXMICROBIOME_SMPL_T524-EndoITSFR and CAM_PROJ_ATRIPLEXMICROBIOME_SMPL_T524-NLB4) were clustered to consensus sequences as described above. Consensus sequences less than 300 bp were removed, as were sequences with BLASTn homology to plant sequences. The remaining sequences were aligned with clone sequences and selected BLASTn matches using MUSCLE v3.8.31 [44]. The resulting alignment was trimmed to a conserved 266 base pair region, and sequences that failed to span the entire length (sequences from ENDOITS PCR products that were only slightly longer than 300 bp) were removed to create a final alignment containing 60 sequences. This alignment was subjected to Bayesian analysis using Mr. Bayes v. 3.1.2 [45]. Specified priors included a nucleotide substitution rate of 6 and an inverse gamma distribution. Four runs utilized randomly initiated Monte Carlo Markov chains for 500,000 generations with a sampling frequency of 100 generations. The basidomycete sequence representing a *Cryptococcus* species was specified as the outgroup. The independent runs converged on similar log-likelihood scores (average standard deviation of split frequencies for the analysis was 0.028) and identical tree topologies, illustrated in Figure 1.

## Results

### Microscopy

**Figure 2:**
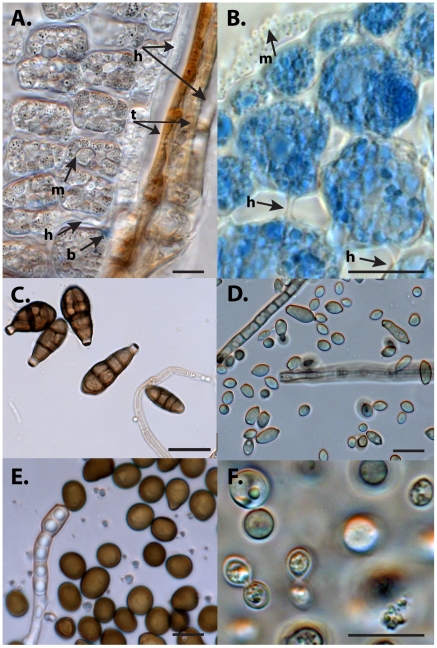
Endophytic fungi associated with *A. canescens* seeds before (A) and during (B) germination. A–B are stained with lactophenol cotton blue. C–F are unstained. A. A mature *A. canescens* seed prior to imbibition reveals a two-layered testa (t) associated with hyaline hyphae (h) on either side. Blue staining regions (b) are rare and indistinct. Embryonic plant cells are covered with dark, spore-forming microbial cells (m). B. A transverse section of an *A. canescens* embryo 48 hours into germination. Spore forming microbial cells (m) are visible on and around plant cells. Intracellular hyphae are visible, but often remain clear (h). C. Group 1 fungi (cf. *Alternaria sp.*, HM596870) had dark brown, ovoid to obclavate conidia separated by both cross and longitudinal septa. D. Group 2 Fungi (cf. *Cladosporium sp.*, HM596871) had brown, one and two celled ovoid and lemon-shaped conidia, some of which formed simple chains E. Group 3 Fungi (cf. *Phoma sp.*, HM596868) had dark round conidia. F. Group 4 fungi (cf. *Cryptococcus sp.*, FJ210546) were single celled, ovoid, and encapsulated. Scale bars  = 10 microns.

Light microscopy revealed hyaline hyphae associated with both the seed coat and the intracellular spaces of the embryonic radicle cells, which were covered with rapidly dividing, yeast-like microbial cells containing dark, spore-like structures (Figure 2A). Stain was poorly absorbed prior to germination, but 48 hours after imbibing water and incubation, blue staining areas dominated the microbial cells associated with plant cell surfaces (Figure 2B). Radicle cross sections revealed microbial cell clusters abundant on the surfaces of plant cells, and sometimes clustering along the outer edges of the embryo (Figure 2B). Microbial isolates observed with light microscopy (Figure 2C–E) are described under isolation of culturable microbes, below.

**Figure 3:**
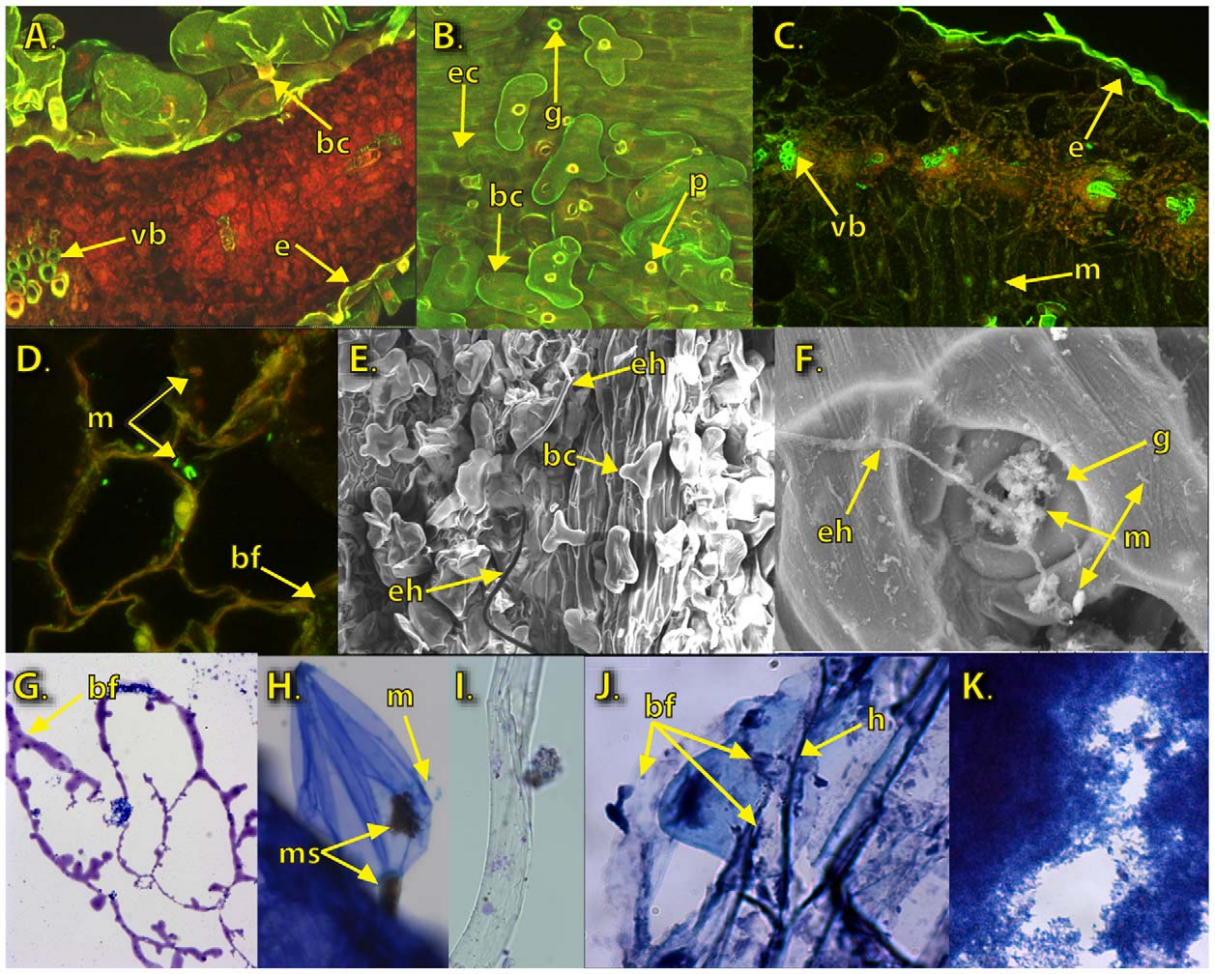
Microbial cells observed on or above sections of micropropagated, regenerated *Atriplex* tissues. Images represent sections excised from leaf (A–F, G–I, K) and root (J) of *Atriplex canescens* (A–B, E, G–K) and *A. torreyi* var *griffithsii* (C,D,F). A. A cross section of regenerated shoots stained with SYTO 9 and propidium iodide. Bladder cells (bc), epidermal cells (e), and cells within vascular bundles (vb) fluoresce green. Scale bar  = 100 µM. B. Leaf surfaces reveal zones of elongated cells (ec) interspersed between dense regions of bladder cells (bc) which penetrate the epidermis through haustoria-like stem cells, creating yellow-collared penetration points (p). Guard cells (g) surrounding the stomatal pores fluoresce green. Scale bar  = 100 µM C. Syto 9 and propidium iodide-stained cross sections of *A. torreyi* reveal fewer, less developed bladder cells than are observed on *A. canescens* leaf surfaces (2A), but shows a similar pattern of Syto9 stain associated with microbes (m) scattered throughout the leaf section and concentrated near the epidermis (e) and around the vascular bundles.(vb) Scale bar  = 100 µM. D. Examination of mesophyll region shown in 2C reveals Syto 9- (green) and propidium iodide (red) stained microbial cells (m) associated with a viscous, biofilm-like residue (bf) that is concentrated near red fluorescing plant cell walls. Scale bar  = 10 µM. E. A scanning electron micrograph (SEM) of a leaf surface of micropropagated *Atriplex*. Bladder cells (bc) are interspersed with regions of long, narrow, surface cells. An, elongated hyphae (eh) extends above the bladder cells to the left of this region. Scale bar  = 100 µM. F. SEM of an *A. torreyi* stomatal complex, An elongated hyphae (eh) extends across the stoma and pore. Microbial cells (m) of varied shapes and sizes are clustered within the pore and on the surfaces of surrounding guard cells (g). Scale bar  = 10 µM. G. A 2 µM section excised from above the leaf surface contains a biofilm-like residue (bf) that corresponds to the intracellular regions of the underlying leaf. Scale bar  = 10 µM. H. A toluidine blue-stained, developing bladder cell on the edge of an *A. torreyi* leaf surface contains melanized microbial cells resembling microsclerotia (ms) in the basal stem and the expanding bladder region. Superficial microbial cells (m) are also visible. Scale bar  = 10 µM. I. A 2 µM section excised from above the leaf surface reveals a single fungal hyphae. Scale bar  = 10 µM. J. An 8 µM thick, trypan blue stained section of regenerated *A. canescens* root reveals a microbial biofilm like residue (bf) containing both hyphae (h) and microbial cells. This residue, which covers all cells, is most visible where it has been slightly raised by the growing tip of a lateral root initial. Scale bar  = 10 µM. K. A 2 µM section excised from above the surface of an *A. torreyi* leaf reveals clusters of trypan blue stained, yeast like cells. Scale bar  = 10 µm.

Leaf sections viewed by confocal microscopy revealed Syto 9 (green) staining of cells surrounding the epidermis and the vascular bundles (Figure 3A–D). Syto9 rapidly penetrates either intact or compromised cell membranes, but is quenched by propidium iodide, which fluoresces red and cannot penetrate uncompromised cell membranes. Both dyes intercalate with DNA. Propidium iodide is also used to stain plant cell walls [46], [47]. This makes the two dyes useful for detection of live microbial cells, and for the detection of microbial biofilms in a variety of plant species [48]. In Atriplex shoots, green fluorescence was observed throughout the bladder cells and epidermis of both *A. canescens* (Figure 3A–B) and *A. torreyi* (Figure 3C and 3D). Cross sections (Figure 3A, 3C–D) revealed additional green fluorescence in vascular bundles (Figure 3A, 3C), and isolated microbial cells dispersed among the mesophyll cells (Figure 3D). The penetration points at which bladder cells attached to or entered the leaf appeared as pores surrounded by yellow collars. These pores were distinguishable from stoma, which exhibited green fluorescing guard cells and were not associated with bladder cells. Bands of elongated hyphae were interspersed between regions of more densely packed bladder cells. These regions were also visible on electron micrographs (Figure 3E), on which loose, filamentous hyphae can be seen clearly separated from the bladder cells. Close up views reveal stomatal regions heavily colonized by yeast-like microbes (Figure 3F), while individual rod shaped, coccus, and irregular-shaped microbial cells can be detected on the surfaces of plant and fungal cells.

Light micrographs of root sections (Figure 3J) exhibited abundant surface hyphae and biofilm-like residues, which were sometimes displaced by emerging lateral root initials. Cryosections removed from above leaf surfaces (Figure 3H–2J) reveal occasional dense clusters of microbial cells (3H, 3J) and hyphae (3I) which may lie above (3H, 3I) or between (3J) plant cells. The larger, more uniform plant cells tended to bend away from the cryostat blade so that microbial cells could be separated from the uppermost intracellular spaces. Plant cell layers would appear in deeper sections.

### Isolation of Culturable Microbes

A total of fifteen isolates were isolated from *A. canescens* seeds following excision from the utricle and surface disinfestation. These were placed in four groups based on morphology. Seven isolates with dark brown, ovoid to obclavate conidia separated by both cross and longitudinal septa were placed in Group 1 (Figure 2C). These microbes were originally isolated on MEA+1.5S, MEA+3.0S, MEA+3.0 NaCl, 0.1% Potato Dextrose Agar, and Nutrient Agar, suggesting tolerance for a broad range of nutrient levels and salinity. Phylogenetic analysis of ITS sequences placed the representative isolate in a clade with various Pleosporales (Figure 1). Group 2 fungi (Figure 2D) included only a single, fast growing strain isolated on MEA +3.0 NaCl. This isolate had brown, one and two celled ovoid and lemon-shaped conidia, some of which formed simple chains. Phylogenetic analysis placed this isolate in a clade with Cladosporium (Figure 1). Group 3 (Figure 2E) included a single isolate with dark, round conidia that grew quickly and was isolated on PDA. This isolate did not group tightly with known genera in the phylogenetic analysis. (Figure 1). Group 4 (Figure 2F) contained six isolates that produced yeast-like conidia. These were isolated on Nutrient agar, MEA+3.0S, or MEA+1.5% NaCl. Although isolated on different media formulations, isolates from all four groups grew satisfactorily on MEA Phylogenetic analysis placed this isolate near the basidiomycete genera, Cryptococcus, which was used as an outgroup (Figure 1).

### Sequences obtained from clones

Fungal sequences obtained from clones isolated from micropropagated plants were deposited in GenBank under accessions FJ601837, FJ601839, and FJ601841-42. Sequences obtained from clones of the isolates included HM195297 and HM596872 (Group 1), HM195296 and HM596871 (Group 2), HM596868 (Group 3) and HM195295 and HM596870 (group 4). Sequences HM596873-6 and HM998754 represent ITS regions of uncultured fungi amplified from total DNA isolated from the micropropagated plants. Table 1 indicates the primers used and the source of the template DNA used for each sequence accession.

### Bacterial Diversity

bTEFAP analysis of 16S rDNA produced 9,294 sequences from *A. canescens* and 15,151 sequences from *A. torreyi* callus that met quality screening criteria. The average read length was 475 bp. BLASTn comparisons to the curated bacterial database revealed diverse sequences with greater than 95% similarity to bacteria from three phyla: Bacteriodetes, Firmicutes, and Proteobacteria. Sequences characteristic of Bacteriodetes (*Bacteriodes*), were only observed in low levels in *A. torreyi*. Firmicutes, which include many gram positive, spore forming species, and insect gut symbionts were represented in *Atriplex* by sequences homologous to *Geobacillus* (a genus associated with thermophiles), *Clostridium* and *Sporobacter* (two genera known for sulfur reducing activity) and *Staphylococcus*, which was particularly abundant in *A. canescens*. Proteobacteria sequences bore homology to the nitrogen fixing genera *Beijerinckia* and *Rhizobium*, to *Sphingomonas*, *Caulobacter,* and the commensal genus, *Escherichia*. Many sequences (68.3% from *A. canescens* and 99.95% from *A. torreyi*) produced alignments with less than 95% similarity to any bacterial species in the database. The vast majority of these sequences were 70–90% similar to one of 9 genera of cyanobacteria, suggesting these may have been derived from amplification of organelle 16S sequences. To refine classification of these sequences, quality screened 16S reads were clustered using CD-HIT-454s. CD-HIT 454 reduces duplicate and nearly identical sequences from 454 datasets to a single consensus sequence identical to the longest read within specified parameters of similarity. Default parameters were modified by changing the sequence identity threshold to 0.95. The output file contained thirty two sequences, which were classified using the RDB classifier [40]. Eleven of these sequences were classified as chloroplasts, and eleven as known bacterial taxa belonging to the phyla shown in Table 2. Ten sequences were unclassified. BLASTn alignments of the unclassified sequences against the GenBank nonredundant nucleotide database indicated these were most likely mitochondrial sequences. The apparent diversity among detected organelle sequences invites further analysis, but is beyond the scope of the current study.

### Fungal Diversity

The fungal isolates described above and illustrated in Figure 2 provided initial evidence that the seed borne fungal community is diverse. Cloning and sequencing of PCR products amplified from total plant DNA provided minimal support for this observation, since sequences obtained from the clones frequently represented plant DNA (Table 1). Only two fungal sequences were obtained from clones, and neither of these were homologous to sequences obtained from isolates (Figure 1). Phylogenetic analysis of clones obtained from cultured and uncultured fungi, and from fTEFAP analysis indicates that different ITS primers revealed different patterns of fungal diversity. Although the high throughput achieved with fTEFAP, uncovered the most diverse array of sequences, there was little overlap between the taxa amplified with ENDOITS forward and reverse primers (Figure 1, green) and the 58A1F and NLB4 primers (Figure 1, red). Although the sequences produced with the latter primer pair produced high scoring pairwise alignments to more taxa (Table 3), amplicons produced with the ENDOITS primers were more genetically diverse, spanning broader clades (Figure 1). Sequences from group 3 and group 4 isolates, were not well represented among the fTEFAP sequences, shown in red and green on Figure 1. Sequences of uncultured endophytes obtained from *A. torreyi* (Figure 1, orange) partitioned into clades distinct from those containing A. *canescens* endophytes.

**Table 3:** Fungal taxa detected in *A. canescens* using fTEFAP analysis of DNA extracted from micropropagated callus.

ID	58A1F-LB4	EndoITS_F&R
Exserohilum	0	0.26
**Dothioraceae**	**0.02**	**0.08**
Leotiomyceta	0.12	0
Fusarium	0	0.04
Cladosporium	10.52	0
Pseudofusarium	0	49.85
Hyalodendriella	2.4	0
Alternaria	0	46.84
Dothideomycete 1	0.06	0
Ochrocladosporium	0.43	0
Capnodiales	0.04	0
**Aureobasidium**	**79.29**	**0.6**
mitosporic Pleosporaceae	0	0.04
Sordariomycete	0	0.56
Dothideomycete 2	6.56	0
Atriplex (host plant)	0	1.73
Davidiellaceae	0.57	0

The ID column lists the fungal genus with the greatest similarity to the query sequence. Sequences with more than 95% similarity to sequences representing more than one genera are identified at the most precise taxonomic level that encompasses all matching genera. The numeric values represent the percent of sequences identified with the indicated primer pair which matched each taxonomic ID. Taxa highlighted in bold type were detected with both primer pairs.

Like sequences obtained from clones, fTEFAP analysis of SSU DNA preferentially amplified host plant sequences. Of the ITS region primers evaluated for fTEFAP, only EndoITSF with EndoITSR and 58A1F with NLB4 produced amplicons. These are summarized in Table 3. Sequences with similarity to ten unique taxa were amplified with 58A1F, NLB4 (Table 3). Sequences with similarity to nine taxa were identified with the EndoITS primer pair. Only sequences homologous to, Aureobasidium and Dothioraceae were detected with both primer pairs, and the percent of total sequences represented by each of these taxa varied with each primer pair. For example, sequences homologous to Aureobasidium represented more than 79% of the sequences amplified with 58A1F, NLB4, but only 0.60% of the sequences amplified with Endo ITSF, EndoITSR. Plant ITS sequences were also amplified with the ENDOITS primer pair, representing the third most abundant group of sequences amplified with these primers.

## Discussion

### PCR Based detection of unculturable microbes

It is widely accepted that more than 98% of microorganisms cannot be isolated. It is increasingly recognized that microbial function is largely a community dynamic [49]. Nonetheless, much of our collective understanding of microbial interactions in the environment as a whole, and within plants in particular, is based on twentieth century approaches to microbial analyses. These approaches relied heavily on axenic cultures to reveal significant microbial functions and processes. With the development of PCR technology, ecologist's concerns that microbial diversity was being grossly underestimated were validated. Efforts to focus on unculturable microbes resulted in development of various “universal” primers that targeted ribosomal genes of diverse microbial taxa [50], [51], [52]. These primers reveal diverse microbes undetectable by isolation based techniques, but may fail to selectively amplify only fungal sequences from mixed samples, or to amplify diverse fungi with equal efficiencys [42], [53], [54].

The majority of universal fungal primers utilized in this study to amplify endophyte DNA amplified plant sequences while omitting many endophytes (Table 1). Two approaches were employed to retrieve additional fungal endophyte sequences. The first was the development of the primer pair ENDOITSf and ENDOITSr (Table 1), created by comparing alignments of Atriplex ITS sequences against the ITS sequences of fungal isolates, and identifying potential fungal primers with minimal affinity for Atriplex DNA. Cloned fragments amplified with these primers revealed novel fungal sequences in A. torreyi callus (Table 1). The second approach, based on the assumption that many endophyte sequences were only amplified in low numbers, was to employ high throughput, fTEFAP analysis to detect low copy number sequences. In the initial fTEFAP analysis, the funSSU primers designed to target fungi amplified only Atriplex sequences (Table 1). To improve detection of endophytes, more diverse ITS regions were targeted, and PCR products, rather than total genomic DNA were subjected to fTEFAP. With this approach, two of the four primer pair tested produced informative sequences representing diverse endophytes. Comparing the relative percentages of sequences homologous to specific taxonomic groups detected with each primer pair demonstrates that differences in primer selection can dramatically influence diversity estimates. The ENDOITS primer pair, which was designed to bypass Atriplex DNA, still amplified plant sequences, though at relatively low abundance. The nested amplification described by Martin and Rygiewicz [42] was effective at eliminating plant sequences, but also failed to amplify sequences several fungal taxa that were detected using the ENDOITS primers (Table 3 and Figure 1). Neither primer set amplified sequences similar to basidiomycetes, even though ITS and SSU sequences from the group 4 isolate (HM596870 and HM195295) clearly align with basidiomycetes, and the ITS sequence was placed with the basidiomycete outgroup in Figure 1. Group 4 morphotypes represented 40% of the cultivable isolates, so basidiomycetes were expected to comprise part of the consortium. Just as the Martin and Rygiewicz [42] primers missed sequences amplified by the ENDOITS primers, the ENDOITS primers failed to amplify sequences representing some taxa that were amplified using Martin and Rygiewicz's nested protocol [42]. Figure 1 suggests that the ENDOITS primers amplified more genetically diverse endophyte sequences than the Martin and Rygiewicz [42] primers.

The bTEFAP analysis applied to bacterial diversity was less complex in that the 16S region of bacterial rDNA is less prone to interspecies variation, hence more robust as a universal target for bacterial diversity studies. Because significant bacterial diversity was illustrated with the 16S primers utilized, no effort was made to optimize further. However, it should not be assumed that any single primer pair is capable of amplifying all, or even most sequences representative of diverse microbial populations. The possibility that additional bacteria are present in the consortium has not been examined.

The ability to detect complex microbial taxa by PCR will always be limited by primer specificity, differences in extractability of DNA from different types of microbial cells, and differences in target sequence abundance. For uncultured microbes, these challenges can be particularly overwhelming, since a system must be well described before it is possible to make refined decisions about which species to target. With these limitations in mind, researchers must balance the desire to maximize detection of diverse species with the time and resources available for analysis. The *in vitro* habitat dramatically reduces the complexity of microbial communities, and may offer a plausible tool for accelerating development of technologies needed for advancing understanding of plant-microbe interactions. As costs of sequencing and genome assembly fall, the potential to sequence and assemble all plant-and-microbial genes extracted from a single *in vitro* microbiome could represent a timely challenge for genomics and computational sciences. Findings would greatly advance current understanding of microbial interactions important to plant biology.

### Fungal Diversity

Microscopic analysis of micropropagated plant tissues revealed fungal hyphae and yeast-like cells associated with roots, leaves, and cryosections excised from phylloplane surfaces (Figure 3). All the microbial isolates obtained from seeds represented fungal taxa. No fungi were isolated from the micropropagated plants, but fungal hyphae were detected with microscopy, and sequences homologous to fungi were identified. The most informative estimates of fungal diversity in the in vitro plant came from the sequences representing uncultured fungi.

Confocal microscopy of leaf surfaces (Figure 3A) revealed bladder cell structures exhibiting green fluorescence typically associated with microbial cells, and haustoria-like penetration points on the leaf surface. Light microscopy (Figure 3H) revealed melanized microbial cells resembling microsclerotia clustered within a developing bladder cell. These observations provide intriguing, albeit inconclusive support for the observation by Barrow et al. [16]that bladder cells on the leaf surface of Atriplex are comprised of fungal endophytes. Bladder cells contribute to host halotolerance, and are generally perceived to originate in the plant [55]. An endophyte that confers salt tolerance could be of significant value, particularly if it could be transferred to alternate hosts. Hence the composition of Atriplex bladder cells merits further investigation.

### Bacterial Diversity

Microscopy reveals numerous small microbial cells embedded within biofilm like formations on the leaf surface (Figure 3F, 3J) or between epidermal cells (Figure 3G) likely originating from cryptic bacteria associated with the embryo (Figure 2B). While the image in Figure 3K resembles a yeast-like colony the exopolymeric matrix, typical of a biofilm, is clearly visible in the intercellular space between the leaf surface cells visible in Figure 3J. Similar formations were observed between the leaf mesophyll cells (Figure 3D). Evidence collected from naturally colonized leaves in open environments, indicates phyllosphere biofilms tend to harbor a wide range of bacterial and fungal organisms [56]. Biofilms may offer competitive advantages to the participating microorganisms by modifying the immediate environment, consequently enhancing individual survival in unfavorable environmental conditions [57]. Such biofilms frequently occur in microniches, either around natural gas and water exchange sites, such as stomata, or along fissures or intercellular spaces that may allow microbial access to carbon compounds and moisture [58]. Phyllosphere biofilms often extend into intracellular spaces within plant tissues [59]. Most previous reports tend either to not specify the origin of the microbial species participating in biofilm consortia, or assume that the main route of acquisition is by recruitment of microbes from the environment. The discovery of seed borne microorganisms that have the capacity to form biofilms begs the question of their possible role in the initiation of endophytic or epiphytic biofilms.

Presence of endophyte species detected by bTEFAP (Table 2) in asyptomatic plant tissues may suggest symbiotic, commensal, or possibly latent pathogenic interactions. In some circumstances, plants may even serve as sinks for human or animal pathogens, including certain *E. coli* and *Staphylococcus* strains [60]. Symbiotic interactions may be hypothesized for the identified diazotrophes (*Beijerinckia*, *Rhizobia*, *Sphingomonas*, *Caulobacter*, and possibly diazotrophic *Clostridium* species), since plants would likely benefit from internal supplies of fixed nitrogen. The range of putatively diazotrophic organisms and the difference in their abundance between the two *Atriplex* species suggests the possible commonality of diazotrophic activity among *Atriplex* endophytes.

### Significance of *in vitro* endophyte consortia

Endophytic associations with diverse species of healthy plants have been recognized since the late 1800's [61], [62]. However, their cryptic nature has limited recognition of the prevalence of such microbes such that *in vitro* plant cultures have historically been perceived axenic until proven otherwise. Natural products extracted from plants *in vitro* are typically assumed to be plant products.

Improvements in detection and analytical capabilities are amplifying the rate at which such perceptions are challenged. It is now reasonable to argue that systemic plant colonization by asymptomatic microbes is the norm, rather than the exception [53], [63], [64], [65], [66]. The revelation that even under aseptic conditions, plant growth and metabolism is influenced by complex interactions with entire communities of cryptic microbes raises new challenges for biochemical, evolutionary, and “-omic” (genomic, transcriptomic, metabolomic…) endeavors as it becomes increasingly evident that molecules isolated from aseptically cultured plants are not necessarily produced by plant cells [67]. New standards for validating plant science research efforts will mandate increased use of systems and metagenomic approaches for examining plant growth, adaptation, and response to the environment in order to expand understanding of the factors regulating host-symbiont interactions *in planta*.

Such efforts are not without promise. In an era marked by global efforts to sustainably increase food and energy production as populations expand and climate changes, improved understanding of microbial interactions that influence plant adaptation could provide technological breakthroughs with which to address these challenges. Evidence that plant-associated microbes may fix atmospheric nitrogen, solubilize soil nutrients, and synthesize natural products that protect host plants against pest and disease has already lead to a steadily expanding industry in biofertilizers [68]. Yet the successful application of microbial inoculants (biofertilizers) to date has been plagued by inadequate understanding of complex interactions between hosts and microbes, and between microbes [69].

The seed borne nature of the *A. canescens* endophyte community demonstrates potential for a diverse community to co-evolve with the host, mandating deeper analysis of the mode by which individual endophytes are transmitted to progeny. Both vertical and horizontal transmission have been reported in seed borne fungi associated with other host plants [70], [71], [72] Although horizontal transmission appears to be more common, [73], vertically transferred taxa may have more significant ecological consequences [18], [20]. Variation in the degree of vertical transmission success is also common [74], and it is conceivable that some endophytes may be transferred by both mechanisms.

The array of microscopic and molecular methods applied in this study provided clear evidence that the associated microbial community was both abundant and diverse, illustrated limitations of individual PCR primer pairs, and demonstrated the utility of high throughput TEFAP methods for detecting rare amplicons within PCR products. TEFAP analysis using the primer pairs which produced diverse microbial sequences in the current study represents a feasible, albeit limited approach for detecting cryptic bacterial and ascomycete endophytes within plant matrices. This approach may be helpful for assessing endophyte transfer during co-cultivation [53].
